# Identifying Depression in Parkinson's Disease by Using Combined Diffusion Tensor Imaging and Support Vector Machine

**DOI:** 10.3389/fneur.2022.878691

**Published:** 2022-06-20

**Authors:** Yunjun Yang, Yuelong Yang, Aizhen Pan, Zhifeng Xu, Lijuan Wang, Yuhu Zhang, Kun Nie, Biao Huang

**Affiliations:** ^1^Department of Radiology, The First People's Hospital of Foshan, Foshan, China; ^2^Department of Radiology, Guangdong Provincial People's Hospital, Guangdong Academy of Medical Sciences, Guangzhou, China; ^3^Department of Neurology, Guangdong Provincial People's Hospital, Guangdong Academy of Medical Sciences, Guangzhou, China

**Keywords:** machine learning, support vector machine, diffusion tensor imaging, Parkinson's disease, depression

## Abstract

**Objective:**

To investigate white matter microstructural alterations in Parkinson's disease (PD) patients with depression using the whole-brain diffusion tensor imaging (DTI) method and to explore the DTI–based machine learning model in identifying depressed PD (dPD).

**Methods:**

The DTI data were collected from 37 patients with dPD and 35 patients with non-depressed PD (ndPD), and 25 healthy control (HC) subjects were collected as the reference. An atlas-based analysis method was used to compare fractional anisotropy (FA) and mean diffusivity (MD) among the three groups. A support vector machine (SVM) was trained to examine the probability of discriminating between dPD and ndPD.

**Results:**

As compared with ndPD, dPD group exhibited significantly decreased FA in the bilateral corticospinal tract, right cingulum (cingulate gyrus), left cingulum hippocampus, bilateral inferior longitudinal fasciculus, and bilateral superior longitudinal fasciculus, and increased MD in the right cingulum (cingulate gyrus) and left superior longitudinal fasciculus-temporal part. For discriminating between dPD and ndPD, the SVM model with DTI features exhibited an accuracy of 0.70 in the training set [area under the receiver operating characteristic curve (ROC) was 0.78] and an accuracy of 0.73 in the test set (area under the ROC was 0.71).

**Conclusion:**

Depression in PD is associated with white matter microstructural alterations. The SVM machine learning model based on DTI parameters could be valuable for the individualized diagnosis of dPD.

## Introduction

Parkinson's disease (PD) is the second most common neurodegenerative disease (1). Parkinson's disease is a heterogeneous disease and can cause several motor and non-motor symptoms. In general, PD can be characterized by the four non-motor symptoms of depression, sleep, olfaction, and cognition disorders, and the four motor symptoms of tremor, rigidity, bradykinesia, and postural instability (2). Depression is the most common non-motor disorder and affects 40–50% of patients with PD (3). Depression in patients with PD is closely related to poor quality of life (4), such as altered mood, cardiovascular sympathetic dysfunction, sleep disturbance, and even active suicidal ideation (5–7). Early diagnosis of depression in patients with PD can help improve their quality of life; however, the pathophysiological mechanism of depression in PD is poorly understood.

Diffusion tensor imaging (DTI) can quantitatively evaluate microstructural alterations of white matter (WM) tracts in vivo (8). Fractional anisotropy (FA) and mean diffusivity (MD) are the two widely employed DTI parameters that are sensitive to detecting microstructural alterations in WM tracts (9). The evidence from DTI studies has shown that there are altered WM tracts, such as in limbic systems, in patients with depressed PD (dPD) (10). Manual tracing of the region of interest (ROI) is the most widely used DTI approach to explore the pathogenesis of dPD (10–12). Diffusion tensor imaging based on the manual ROI method has shown decreased FA values in the anterior cingulate bundle in patients with dPD compared to patients with non-depressed PD (ndPD) (10). The decreased FA values were also found in the bilateral mediodorsal thalamic regions in patients with dPD (11). However, most prior DTI studies based on manual tracing of ROI measurements are not able to comprehensively evaluate the entire WM regions in patients with PD. The atlas-based whole-brain WM analysis (ABA) is an automated and convenient DTI analysis approach that can effectively detect the integrity of the whole-brain WM (13). Therefore, it can quantitatively assess the microstructure of WM tracts and ameliorates possible measurement errors associated with the manual ROI method (14). Recently, the ABA method has become a widely used method in DTI studies of nervous system diseases (15–17).

At present, the most effective diagnostic criterion of dPD is based on neuropsychological examinations but often shows some clinical challenges and limitations (18). Currently, clinical physician recognition in dPD is not enough. More than 60% of self-reported patients with dPD were not recognized by neurologists on the Unified Parkinson's Disease Rating Scale (UPDRS) (19). However, one study showed that the incidence rate of depression is substantially elevated in the early stage of PD (20). In clinical practice, the current diagnostic gold standard for depression is the standardized clinical interview according to diagnostic criteria (such as DSM-V), while in non-research settings, this diagnostic tool is impractical for detecting depression in PD (18). Consequently, many scoring scales (either informant-rated, self-report, or clinician-based) are usually used to screen mental symptoms; however, it is unclear which of these screening tools for the detection of depression is most accurate (21). Therefore, it is crucial to develop a more accurate approach to identifying dPD. For years, machine learning has been widely used to build classifiers for medical diagnosis. The support vector machine (SVM) is a well-known algorithm with good generalization performance and robust classification ability for machine learning models (22). Based on the DTI technique, the SVM classifier has successfully been applied to automate discrimination in the different categories (such as patients and normal controls) in many studies, and the SVM analysis was demonstrated with high performance (23, 24). To our best knowledge, there are no studies that have used the SVM method based on DTI data to discriminate the patients with dPD from those with ndPD.

The purpose of this study was to conduct whole-brain DTI data analyses using the ABA approach to explore WM microstructural changes in patients with dPD. In addition, SVM was trained with FA and MD values of different WM tracts to determine the probability of discriminating dPD from ndPD.

## Materials and Methods

### Study Design

We recruited 37 patients with dPD and 35 patients with ndPD from our institution from July 2014 to December 2019, and 25 healthy control (HC) subjects matched for age and gender were also enrolled. All patients with PD were diagnosed according to clinical diagnostic criteria from the UK Parkinson's Disease Brain Bank by two experienced physicians (25). Depression was diagnosed according to the Diagnostic and Statistical Manual of Mental Disorders 4th edition (DSM-IV) criteria (26) by a clinically experienced psychiatrist. None of the patients were receiving antidepressant medications, and all patients were without typical motor symptoms. Motor severity of PD was assessed by the Unified Parkinson's Disease Rating Scale part III (UPDRS-III) (27) and the modified Hoehn and Yahr (H-Y) scale (28), respectively. The degree of depression was assessed by the 24-item Hamilton Depression Rating Scale (HAM-D) score and anxiety was assessed by the Hamilton Anxiety Rating Scale (HAMA) (29). The general cognitive dysfunction was assessed by the Mini-Mental State Examination (MMSE) and the Montreal Cognitive Assessment (MoCA) (30). All the PD subjects were right-handed.

Exclusion criteria were as follows: (1) major psychiatric disorders (e.g., schizophrenia, bipolar disorder); (2) history of a brain tumor, cerebrovascular disorders, or brain trauma; (3) obvious encephalatrophy; (4) history of antidepressant medication use; (5) MMSE score <24 or H-Y scale score > 4; and (6) obvious motion artifacts in imaging studies. This study was approved by our Institutional Ethics Committee, and written informed consent was obtained from all participants.

### Magnetic Resonance Imaging Acquisition

Magnetic resonance imaging data of all patients were performed using a 3.0 T MR scanner (Signa Excite HD; GE Healthcare, Milwaukee, WI, USA) with an eight-channel head coil. Any anti-Parkinsonian medications were discontinued 12 h before the imaging studies to decrease the effects of the drug(s) on brain neural activity. All subjects were instructed to not move during the MR scan, and foam padding and earplugs were used to limit head movement and decrease the impact of acoustic noise.

The DTI data were obtained using an echo planar imaging (EPI) sequence with the following scan parameters: Repetition time/echo time (TR/TE) = 15,275/76.7 ms, FOV (field of view) = 320 × 320 mm, matrix = 128 × 128, slice thickness = 2.5 mm with no gaps, 25 different diffusion directions with b = 1,000 s/mm^2^, the total number of the images = 1,560, and scan time = 7 min and 8 s. The Array Spatial Sensitivity Encoding Technique (ASSET) was used to decrease the acquisition time and image distortion.

High resolution, 3D T1-weighted data was obtained using a Fast Spoiled Gradient Recalled Echo Inversion Recovery (FSPGRIR) sequence with the following parameters: TR = 8.4 ms, TE = 3.3 ms, flip angle (FLA) = 13°, matrix = 256 × 256, voxel size = 0.94 × 0.94 × 1 mm, slice thickness = 1 mm, and the total number of the sagittal slices = 146.

### Diffusion Tensor Imaging Data Processing

All DTI data were processed using a pipeline toolbox (PANDA; Pipeline for Analyzing brain Diffusion images, http://www.nitrc.org/projects/panda) based on the MATLAB R2016a program (The MathWorks, Natick, NA, USA). PANDA is a pipeline toolbox for fully automated to perform analyses and calculations of brain diffusion images. The main processing steps include the following: (a) The DICOM data are converted to NIFTI data format; (b) The brain mask is estimated and non-brain tissues are removed; (c) Eddy-current effect and head movement are corrected; (d) The DTI metrics, including FA and MD, are calculated; (e) All of the individual FA images of the native space are registered non-linearly into a FA standard template in the Montreal Neurological Institute (MNI) space using the *fnirt* command of FSL; (f) The data are output for atlas-based analysis. We defined the ROIs on the Johns Hopkins University (JHU) WM tractography atlas (31). The DTI data were processed by PANDA based on the JHU WM atlas is an established method and performed in many studies (14–17). This WM atlas in the standard space may provide better statistical accuracy and sensitivity (31). The JHU WM tractography atlas parcellates the entire WM into 20 ROIs automatically (16).

### Support Vector Machine Classification Model for dPD and NdPD

An SVM was used to discriminate between patients with dPD and patients with ndPD. The prediction model was constructed based on dPD and ndPD and data from the HC group was not used in the model. The SVM model was established based on combinations of different DTI indexes. We ran the non-linear SVM using the Python Sklearn library algorithm (32). All of the data from all patients were randomly divided into a training set (80%) and a test set (20%). The FA and MD values from the 20 ROIs in different WM tracts were chosen as classifier features of the SVM (23). We used a training set for feature selection. Subsequently, based on the DTI data, a grid search algorithm within a 10-fold cross-validation procedure was implemented to automatically identify the suitable tuning parameters for the SVM model. The validation procedure implemented in the SVM was repeated 100 times. The grid search technique helps perform an exhaustive search to obtain specified parameter values (*c* and *g*) for an estimator (33). The performance of the SVM model was assessed by determining the accuracy, sensitivity, specificity, positive predictive value, negative predictive value, and area under the receiver-operating-characteristic curve (AUC) for distinguishing dPD from ndPD. Each feature has different effects on the classification results. In addition, the importance of features was further weighted according to the trained SVM model.

### Statistical Analysis

Statistical analysis was performed using SPSS version 20 software (Chicago, IL, USA). For statistical analysis of any group differences in age, education, and neuropsychological scores, a one-way ANOVA test was applied. Differences in gender between the groups were examined with the χ^2^ test, and the independent sample *t*-test was used to analyze differences in UPDRS-III, H-Y, and PD duration time between dPD and ndPD. The values of *p* < 0.05 were considered to indicate statistical significance. For statistical analysis of DTI features between patients with dPD and ndPD, using UPDRS-III, MMSE, and MoCA scores as covariates. An analysis of covariance (ANCOVA) was used to identify brain areas that had significant differences across the three groups. The *post hoc* analyses were performed to further explore the between-group differences using LSD's *post hoc* tests after ANCOVA analysis with years of education, gender, and age as covariates. A false discovery rate (FDR) was used to correct for multiple comparisons. The DTI features with FDR-corrected *p* < 0.05 after analysis was selected to build the SVM model.

## Results

### Demographic and Clinical Characteristics

The patient's detailed demographic and clinical characteristics are presented in Table 1. The HAM-D score and HAMA score of the dPD group was higher than the ndPD and HC groups (*p* < 0.001), which is consistent with previous studies revealing that dPD often coexisted with anxiety disorder. There were no significant differences in gender, age, years of education, or MMSE, and MoCA among the three groups. There were no differences in disease duration, H-Y scale, and UPDRS-III between the two groups (in all, *p* > 0.05).

**Table 1:** Demographic and clinical characteristics of all subjects.

**Variables**	**dPD (*n* = 37)**	**ndPD (*n* = 35)**	**HC (*n* = 25)**	***p***	***p* (dPD *vs*. ndPD)**
Gender (M/F)	19/18	21/14	9/16	0.185	0.632
Age (year)	60.73 ± 11.22	62.40 ± 11.10	57.08 ± 7.93	0.256	0.789
Education (year)	9.54 ± 4.54	10.00 ± 4.54	8.52 ± 3.57	0.425	0.654
Disease duration (year)	5.01 ± 3.01	3.57 ± 3.65	/	/	0.073
H-Y scale	2.18 ± 0.39	2.17 ± 0.61	/	/	0.972
UPDRS-III	37.46 ± 13.22	32.63 ± 13.10	/	/	0.124
HAM-D	22.95 ± 7.75	5.94 ± 3.24	2.52 ± 2.74	<0.001^*^	<0.001^*^
HAMA	20.00 ± 8.30	6.09 ± 3.27	2.92 ± 2.90	<0.001^*^	<0.001^*^
MMSE	26.65 ± 2.21	27.66 ± 2.04	26.96 ± 2.39	0.148	0.055
MoCA	20.92 ± 4.21	22.60 ± 3.65	22.04 ± 3.43	0.171	0.065

*Data are presented as mean ± SD. For continuous variables, one-way ANOVA was carried out. For categorical variables, χ^2^ tests were carried out. The χ^2^ test was used to analyze the gender among the three groups. One-way ANOVA was used for age, education and neuropsychological scores, and a pairwise post hoc test was subsequently used to identify significant main group differences. The independent sample t-test was used to analyze differences of UPDRS-III, H-Y, PD duration time between dPD and ndPD*.

* *p < 0.05 indicates statistical significance*.

*dPD, PD with depression; ndPD, PD without depression; H-Y scale, Hoehn-Yahr scale; UPDRS- III, Unified Parkinson's Disease Rating Scale scores-III; HAM-D, Hamilton Depression Scale; HAMA, Hamilton Anxiety Scale; MMSE, Mini-Mental State Examination; MoCA, Montreal Cognitive Assessment*.

### Diffusion Metric Changes in Different WM Regions

**Figure 1:**
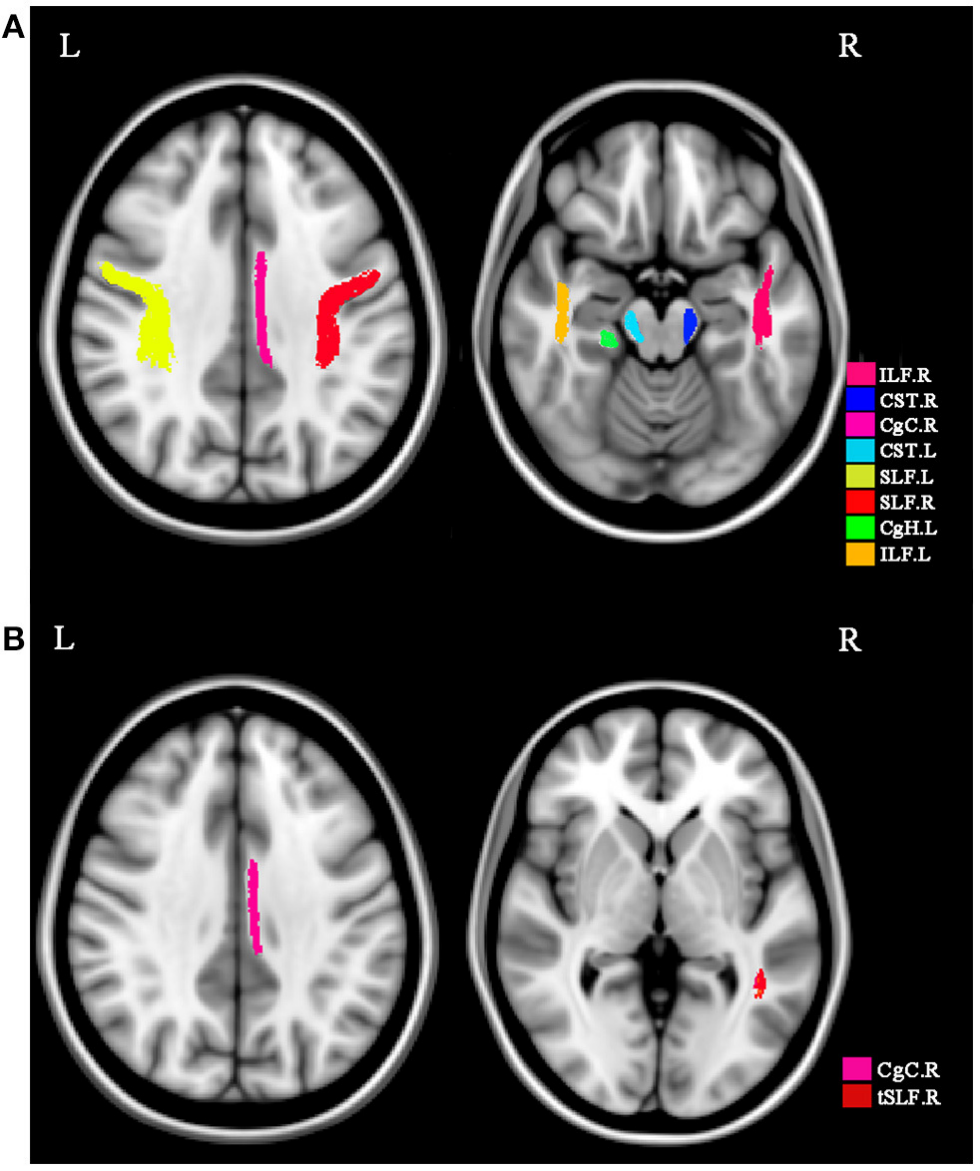
WM regions with significant differences among dPD, ndPD, and HC. Significant WM regions are marked by various colors in the axial planes. **(A)** The FA values of white matter tracts with significant difference among groups. **(B)** The MD values of white matter tracts. These WM regions include CST.L, CST.R, CgC.R, CgH.L, ILF.L, ILF.R, SLF.L, SLF.R, CgC.R, tSLF.R. CST, corticospinal tract; CgC, cingulum (cingulate gyrus); CgH, cingulum hippocampus; ILF, inferior longitudinal fasciculus; SLF, superior longitudinal fasciculus; tSLF, superior longitudinal fasciculus-temporal part; FA, fractional anisotropy; MD, mean diffusivity; R, right; L, left.

We compared the changes in FA and MD among three groups using ABA analysis. The *post hoc* comparisons showed significant differences in DTI features between dPD and ndPD with *p* < 0.05 (after FDR corrected) as shown in Figure 1 and Table 2. Compared with the patients with ndPD, the patients with dPD exhibited decreased FA in the bilateral corticospinal tract, right cingulum (cingulate gyrus), left cingulum hippocampus, bilateral inferior longitudinal fasciculus, bilateral superior longitudinal fasciculus, and increased MD in the right cingulum (cingulate gyrus) and left superior longitudinal fasciculus-temporal part.

**Table 2:** Brian WM regions with significant difference among dPD, ndPD, and HC.

**Regions**	**DTI parameters**	**dPD (*n* = 37)**	**ndPD (*n* = 35)**	**HC (*n* = 25)**	***p***	***p* (dPD *vs*. ndPD)**
CST.L.	FA	0.589 ± 0.022	0.573 ± 0.017	0.585 ± 0.020	0.007^*^	0.002
CST.R	FA	0.590 ± 0.019	0.575 ± 0.020	0.584 ± 0.024	0.013^*^	0.004
CgC.R	FA	0.460 ± 0.035	0.440 ± 0.035	0.477 ± 0.036	0.002^*^	0.038
CgH.L	FA	0.457 ± 0.028	0.435 ± 0.027	0.451 ± 0.045	0.021^*^	0.003
ILF.L	FA	0.446 ± 0.020	0.432 ± 0.019	0.449 ± 0.023	0.007^*^	0.001
ILF.R	FA	0.454 ± 0.022	0.441 ± 0.024	0.461 ± 0.027	0.011^*^	0.024
SLF.L	FA	0.376 ± 0.023	0.386 ± 0.017	0.391 ± 0.019	0.013^*^	0.026
SLF.R	FA	0.401 ± 0.020	0.387 ± 0.022	0.405 ± 0.025	0.007^*^	0.016
CgC.R	MD	0.714 ± 0.006	0.736 ± 0.005	0.713 ± 0.007	0.011^*^	0.041
tSLF.R	MD	0.776 ± 0.008	0.806 ± 0.008	0.773 ± 0.009	0.011^*^	0.040

All values are presented as mean ± SD. MD values × 10^−3^ mm^2^/s. Using analysis of covariance (ANCOVA) for the comparison of FA and MD values among the three groups. The pairwise post hoc comparisons were then performed using t-tests (^*^p < 0.05 after FDR corrected).

*dPD, PD with depression; ndPD, PD without depression; HC, health control; CST, corticospinal tract; CgC, cingulum (cingulate gyrus); CgH, cingulum hippocampus; ILF, inferior longitudinal fasciculus; SLF, superior longitudinal fasciculus; tSLF, superior longitudinal fasciculus-temporal part; FA, fractional anisotropy; MD, mean diffusivity; R, right; L, left*.

### Support Vector Machine Classification of dPD and NdPD

**Figure 2:**
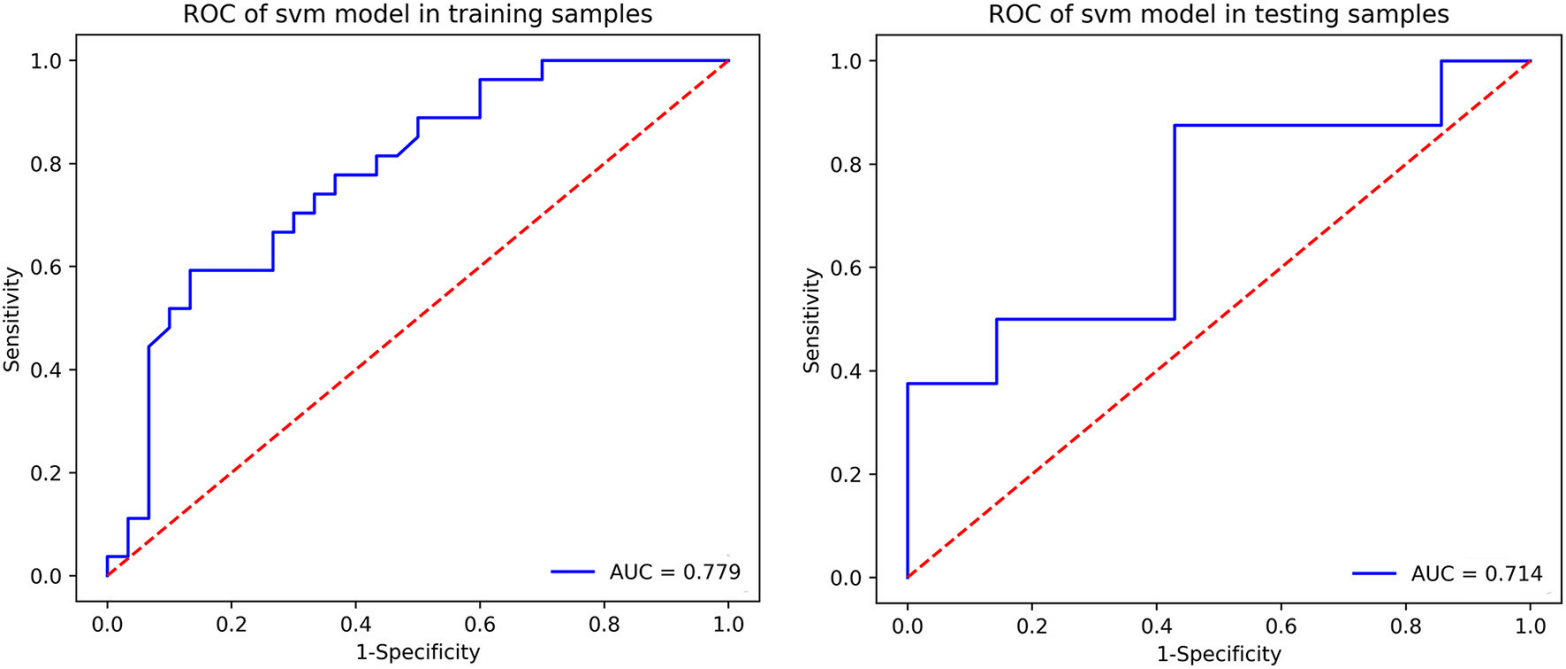
ROC of the SVM model in the training and testing samples.

**Figure 3:**
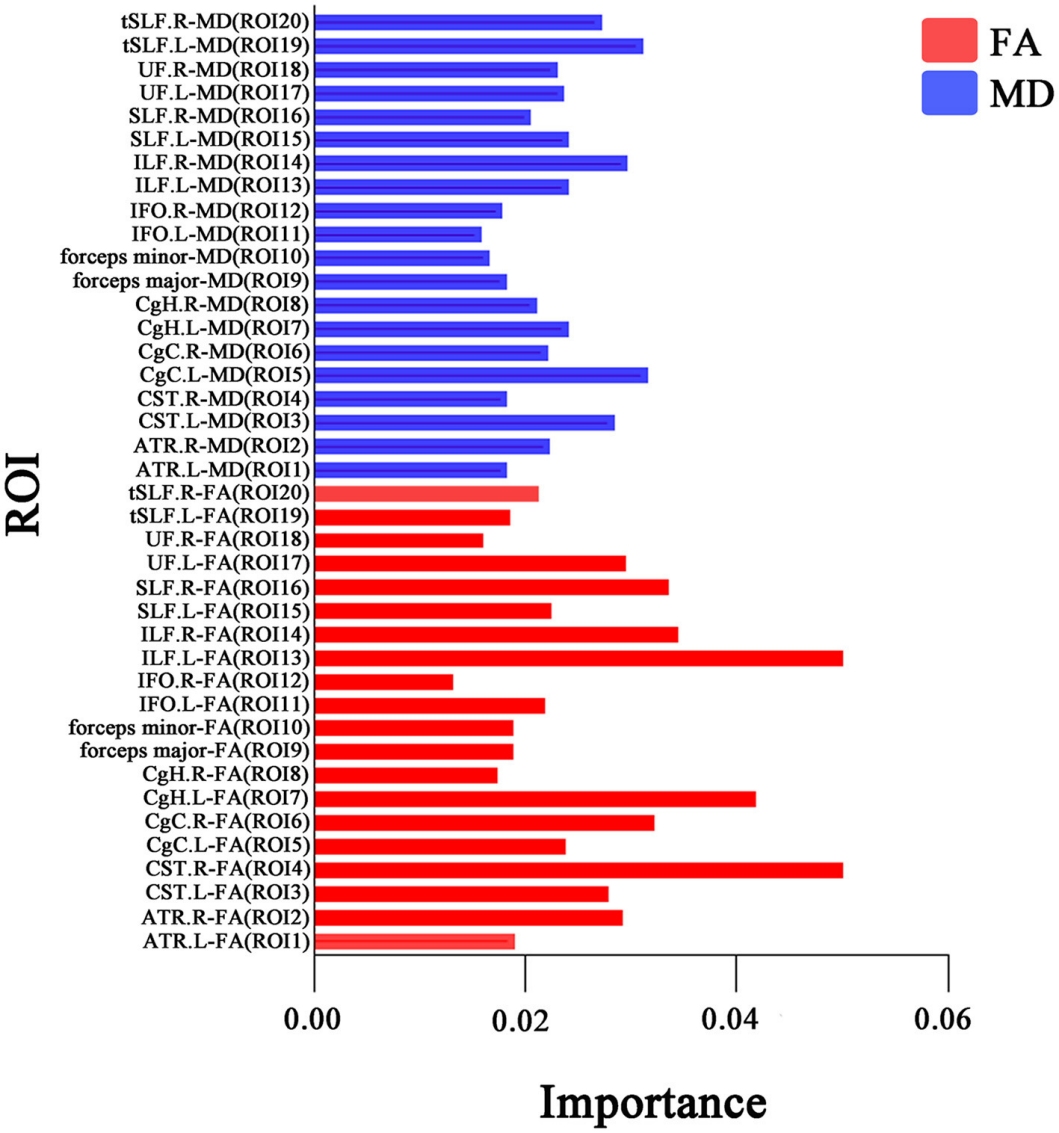
The importance index of the FA (red color) and MD (blue color) values of all white matter ROIs. The longer column represents higher importance. The most important features are the FA in the ILF.L (ROI = 13). ROI, region of interest; ILF, inferior longitudinal fasciculus; FA, fractional anisotropy; MD, mean diffusivity; R, right; L, left.

The prediction summary of the SVM is shown in Table 3 and Figure 2. Using the diffusion metrics (FA, MD) of all of the ROIs as features of the SVM classification model achieved moderate performance in distinguishing dPD from ndPD. Briefly, the SVM model classification of accuracy was 0.70, sensitivity was 0.67, specificity was 0.73, positive predictive value was 0.69, negative predictive value was 0.71, and AUC–ROC was 0.78 in the training set; and accuracy was 0.73, sensitivity was 0.88, specificity was 0.57, positive predictive value was 0.70, negative predictive value was 0.80, and AUC–ROC was 0.71 in the test set. The classification importance of each feature was further examined, and the results of the importance of the various ROIs are presented in Figure 3. The most important features were the FA value along the left inferior longitudinal fasciculus (ROI = 13).

**Table 3:** Prediction outcome summary.

**Model**	**ACC**	**SEN**	**SPE**	**PPV**	**NPV**	**AUC–ROC**
Train (*n* = 57)	0.70	0.67	0.73	0.69	0.71	0.78
Test (*n* = 15)	0.73	0.88	0.57	0.70	0.80	0.71

*ACC, accuracy; SEN, sensitivity; SPE, specificity; PPV, positive predictive value; NPV, negative predictive value; AUC–ROC, area under the receiver-operating-characteristic (ROC) curve*.

## Discussion

In this study, the ABA method was used to examine alterations of WM microstructural integrity in patients with dPD. First, we found WM tract impairment of the limbic system and non-limbic system in the dPD group. Second, we established an SVM machine learning model to classify dPD employing the diffusion metrics (FA, MD) that exhibited the performance of features that had an acceptable accuracy. The findings in this study support the contention that WM damage is a common pathology in dPD, and this may assist in understanding the pathophysiological mechanism of depression in PD. The SVM classification model based on DTI features might promote the individualized diagnosis of dPD.

In the present study, patients with dPD showing microstructural alteration of WM tracts were mainly located in the limbic system. The cingulum is a core structure in the Papez circuit of the cholinergic system that connects limbic and prefrontal cortex regions and plays an important role in emotional regulation and reward behavior (34). The disruption of the cingulum may lead to depression. Our results showed decreased FA in the right cingulum (cingulate gyrus) and left cingulum hippocampus and increased MD in the right cingulum (cingulate gyrus) in the dPD group compared to the ndPD group. A significant reduction in FA value in the cingulum bundle has been demonstrated in depressed patients compared with non-depressed control subjects (35). One morphological study detected that decreased volume in the hippocampus was correlated with depression in patients with PD (36). Reductions in FA value in the cingulum bundle in depressed PD patients compared with non-depressed patients with PD have been observed (37–39). Increased MD value in the cingulum hippocampus has also been found in depressed patients with PD (37, 39). Thus, the above findings suggested that the alteration of the white matter integrity in the limbic systems may contribute to the development of depression in PD.

In this study, we also found microstructural alterations in non-limbic fibers of the brain in patients with dPD, such as decreased FA values in the bilateral corticospinal tract, bilateral inferior longitudinal fasciculus, bilateral superior longitudinal fasciculus, and left superior longitudinal fasciculus-temporal part in patients with dPD. The superior longitudinal fasciculus contains connections between the temporal, occipital, parietal, and frontal lobes and the limbic system, and is an important mediator of mood-related function (40). The inferior longitudinal fasciculus is an association fiber that connects regions of the temporal and occipital lobes and is mainly related to visual processing, object recognition, and emotional regulation (41). One study revealed reduced FA value in superior longitudinal fasciculus and inferior longitudinal fasciculus in depressed PD compared to non-depressed PD (39). An additional study showed decreased FA value in the superior longitudinal fasciculus in depressed patients compared to healthy controls (42). Furthermore, one recent finding support that significantly reduced FA in the corticospinal tract occurs in the severely dPD group when compared with the ndPD group (43). These results suggested the alterations of non-limbic fibers might be involved in the pathogenesis in patients with dPD.

Based on DTI parameters obtained by the ABA method, the SVM classifier exhibited moderate accuracy in classifying dPD and ndPD. SVM machine learning model is a useful machine learning classification algorithm and has been shown to successfully discriminate Alzheimer's disease from normal cognition groups using diffusion metrics as features (23). The present diagnosis of dPD mainly depends on clinical evaluation based on neuropsychological examinations, and its methods may tend to lack diagnostic accuracy (18). Hitherto, none of the studies have combined the DTI and SVM machine learning model in identifying dPD. Thus, the current study used the DTI metrics as the SVM classifier's features and achieved acceptable performance for discriminating dPD from ndPD. One study performed a radiomic analysis approach that extracted features from the resting-state functional MRI for the diagnosis of dPD (44). The prediction accuracy of SVM trained by resting-state functional MRI features was achieved by 0.65 for distinguishing dPD from ndPD. Furthermore, we observed that the FA value of the left inferior longitudinal fasciculus was the most important classification feature in the SVM model.

We recognize some limitations to this study. First, the numbers of patients were relatively small, however, despite the small sample size, we were able to identify the WM microstructural integrity was impaired in patients with dPD. Second, brain abnormalities in some regions could influence the quality of template matching when using the ABA method. Thus, the patients with obvious encephalatrophy identified on conventional MRI scanning sequences were excluded. Finally, our study investigated microstructural changes only in the WM and the depression in PD involved WM and the gray matter, thus it is possible to later combine both WM and gray matter to analyze the disease.

## Conclusion

In conclusion, the present study detected WM microstructural abnormalities in patients with dPD using atlas-based DTI analysis. Depression in PD was associated with white matter microstructural alterations in the limbic and non-limbic systems. Furthermore, the SVM machine learning model based on DTI data had an acceptable accuracy to identify dPD.

## Data Availability Statement

The raw data supporting the conclusions of this article will be made available by the authors, without undue reservation.

## Ethics Statement

The studies involving human participants were reviewed and approved by Ethics Committee of Guangdong Provincial People's Hospital. The patients/participants provided their written informed consent to participate in this study.

## Author Contributions

All authors listed have made a substantial, direct, and intellectual contribution to the article and gave the final approval for the manuscript to be published.

## Funding

The study was supported by the National Natural Science Foundation of China (Grant Number: 82071871).

## Conflict of Interest

The authors declare that the research was conducted in the absence of any commercial or financial relationships that could be construed as a potential conflict of interest.

## Publisher's Note

All claims expressed in this article are solely those of the authors and do not necessarily represent those of their affiliated organizations, or those of the publisher, the editors and the reviewers. Any product that may be evaluated in this article, or claim that may be made by its manufacturer, is not guaranteed or endorsed by the publisher.
